# Denoise-GS: Self-Supervised Denoising for Sparse-View 3D Gaussian Splatting

**DOI:** 10.3390/s26020651

**Published:** 2026-01-18

**Authors:** Yabo Xu, Jin Ding, Jianbin Zhang, Ping Tan, Mingrui Li

**Affiliations:** 1 College of Automation and Electrical Engineering, Zhejiang University of Science and Technology, Hangzhou 310023, China; 222307855020@zust.edu.cn (Y.X.);; 2 School of Information and Communication Engineering, Dalian University of Technology, Dalian 116085, China

**Keywords:** 3D Gaussian splatting, denoise, volume rendering, view synthesis, sparse-view reconstruction

## Abstract

Three-dimensional Gaussian splatting has emerged as a mainstream method in the field of new viewpoint synthesis due to its outstanding performance. However, its generation quality typically degrades significantly when input viewpoints are sparse. The introduction of InstantSplat further improved new viewpoint generation in sparse viewpoint scenarios. Nevertheless, these methods produce suboptimal results in sparse viewpoint scenes with noise and no camera prior. To address this issue, we propose Denoise-GS, a two-round optimization framework combining N2V-UNet denoising with InstantSplat rendering. First, Noise2Void performs self-supervised denoising on the input image. Next, pose grouping is conducted based on InstantSplat rendered results. Finally, a second round of refinement is applied to the UNet through a joint loss function. The final denoised result is then re-rendered to achieve a higher-quality output image. To simulate a real noisy environment, we added Gaussian noise to the input images. Tests on multiple datasets show that, compared with other mainstream methods, our approach produces images with higher PSNR and SSIM. The method performs well in novel view generation when the input images are sparse and noisy, providing an innovative and practical solution for three-dimensional reconstruction.

## 1. Introduction

In recent years, explicit representation-based three-dimensional reconstruction methods have made significant progress in novel view synthesis. Three-dimensional Gaussian splatting (3DGS) represents a scene as a set of differentiable Gaussian distributions, enabling high-quality and efficient 3D rendering. It excels in both real-time performance and visual effects [1,2]. It has made substantial contributions in fields such as SLAM [3], autonomous driving, environmental perception, AR, VR, and more. Compared to traditional implicit neural representations, 3DGS offers faster convergence and better interpretability, thus attracting widespread attention across various application scenarios [4]. However, existing 3DGS methods still face key challenges in practical applications, especially when the input is sparse, resulting in poor reconstruction quality [5].

To alleviate the information deficiency caused by sparse views, several improvement strategies have been proposed, such as introducing stronger geometric priors and improving initialization and optimization processes to enhance reconstruction stability. InstantSplat [6] is a representative work in this area. It combines DUSt3R [7] with 3DGS and, through more reliable 3D initialization and optimization strategies, enables the model to generate high-quality novel view results even under limited viewpoints. However, in real-world acquisition environments, sparse viewpoints often coexist with noisy scenes. For low-light noise and dynamic range limitations, methods like LO-Gaussian [8] and LL-Gaussian [9] have jointly modeled degradation factors within the 3DGS framework to improve novel view quality in extremely low-light scenarios. For complex weather conditions, research has begun to explore visibility restoration and reconstruction combined with 3DGS under conditions such as rain and fog, such as DeRainGS [10], to mitigate the impact of rainfall streaks and fog scattering on geometry and texture optimization. However, these denoising 3DGS methods are designed for specific scenarios and lack generalization. This work focuses on addressing the issue where image noise, caused by external factors, leads to low quality novel view generation in 3DGS. This problem can be mitigated by denoising the images at the input stage.

Early image denoising methods primarily relied on prior-based filtering strategies, such as mean filtering, bilateral filtering [11], and BM3D [12]. These methods generally suppress noise by modeling the local smoothness or similarity of the image, and they can work well in scenes with weak noise or simple structures. However, when images contain complex textures or high noise levels, these methods often over-smooth details, resulting in texture loss. With the development of deep learning, supervised denoising methods, represented by DnCNN [13] and FFDNet [14], have gradually become mainstream. These methods learn the mapping relationship between noisy and clean images, achieving significant results under synthetic noise conditions. However, these methods rely heavily on large amounts of paired clean–noise samples, which are difficult to obtain in real-world scenarios, limiting their practical applications. To mitigate the reliance on paired clean data, researchers have proposed unsupervised denoising methods, with Noise2Noise (N2N) [15] being one of the earliest representative works. N2N utilizes the statistical independence of noise to perform effective denoising without requiring clean labels. However, it still depends on paired noisy samples as input during training, which is often not feasible in real-world scenarios. To further reduce the reliance on paired data, Noise2Void (N2V) [16] was introduced, which employs a blind spot mechanism and allows training using only a single noisy image. By masking the central pixel and predicting it using surrounding context, the model can learn the noise distribution in an unsupervised manner, thus providing greater flexibility and applicability in practical scenarios.

**Figure 1:**
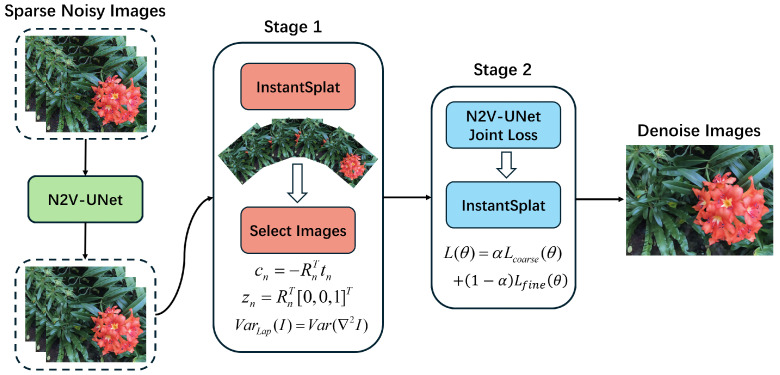
Method overview. This diagram illustrates the entire process from inputting a sparse, noisy image to outputting a denoised image. It undergoes preliminary denoising via N2V-UNet, followed by a first round of rendering and frame selection strategies. Finally, after a second round of joint loss optimization, the denoised image is obtained.

Building on these issues, we propose Denoise-GS. We incorporate the UNet [17] architecture into N2V as the core network structure. The overall framework is shown in Figure 1. Unlike traditional N2V, which only relies on local pixel statistics, we use UNet as the feature extraction framework, allowing the network to leverage both local and global pixel information during unsupervised denoising. Our method combines the unsupervised denoising strategy with InstantSplat and constructs a closed-loop optimization mechanism between denoising and rendering, resulting in higher quality novel view generation in sparse and noisy scenes.

Experiments on multiple datasets show that this joint training strategy can recover fine textures under sparse view and unsupervised conditions. It also produces images that are more natural and consistent across multiple views. Using PSNR, SSIM, and LPIPS as evaluation metrics, Denoise-GS suppresses noise effectively while better preserving the detailed textures of the original scene. Compared with directly applying existing 3DGS methods and their representative improved variants, Denoise-GS shows higher stability. Ablation experiments also confirm that removing any single component leads to a drop in stability and detail performance.

The main contributions of this paper can be summarized as follows:

(1) We developed a two-round sparse viewpoint and noisy image denoising 3DGS framework comprising three core methods: N2V-UNet, pose-based frame selection strategy, and joint loss. This enables it to generate superior results even under constraints of limited viewpoints and input images with significant noise.
(2) Our method achieves higher PSNR and SSIM scores across diverse scenarios while more reliably preserving fine texture details. It effectively mitigates texture blurring and color instability caused by noise, validating the practicality and effectiveness of the proposed framework.

## 2. Related Work

This section reviews three research objectives relevant to this paper: explicit 3D representations and differentiable rendering for new viewpoint synthesis, image denoising learning for real-world noise, and 3D reconstruction methods under sparse viewpoints and degenerate conditions. We highlight key advancements in efficiency, robustness [18,19,20], and multi-view consistency [21,22] of focus methods, while pointing out their limitations in unlabeled noise adaptation and closed-loop optimization. This establishes the theoretical foundation for the joint denoising and reconstruction framework proposed in this paper. Simultaneously, we will survey representative advancements relevant to our system, such as supervised and unsupervised denoising algorithms and sparse viewpoint reconstruction, and explore their relationship with our approach.

### 2.1. Three-Dimensional Gaussian Splatting

NeRF [2] was proposed in 2020. It uses a neural network to learn mapping from spatial coordinates to color and density, and has made important progress in multi-view synthesis. However, its rendering cost is high and its training time is long. This limits its use in complex scenes and real-time applications. To address this problem, 3D Gaussian splatting (3DGS) uses an explicit three-dimensional Gaussian representation and differentiable rasterization. It represents the scene with a finite set of Gaussian primitives and achieves real-time novel view synthesis while maintaining high reconstruction quality. Later, Mip-Splatting [23] reduces aliasing by multi-scale Gaussians and anti-aliased projection, and Scaffold-GS introduces a geometric scaffold to constrain the Gaussian distribution and the optimization process, which improves detail and convergence in complex viewpoints and large scenes. In addition, a series of later works [24,25,26] extend the representation ability and application range of 3DGS through structural priors, sampling strategies, and optimization acceleration, making Gaussian splatting more versatile for real-time rendering and 3D reconstruction.

However, the above methods are still sensitive to image degradation. Sensor noise, motion blur, camera pose errors, and inconsistencies in exposure and color can all be amplified in the reconstruction results. This is more likely to cause texture blur and local artifacts under sparse views and noisy conditions. To reduce these problems, some works [27,28,29] explicitly model the blur kernel or noise distribution during 3DGS optimization. They introduce degradation-aware losses or regularization terms in the volume rendering or Gaussian projection stage and combine them with specific degradation priors or imaging models to constrain the reconstruction process. In this way, they improve reconstruction quality in degraded scenes under given degradation assumptions.

Overall, most of these improvements adapt to image degradation by explicit noise or blur modeling inside 3DGS, and they depend on the imaging model and the data acquisition process. In contrast, this paper does not redesign a noise model inside the main 3DGS framework. Instead, we introduce an external unsupervised denoising network and optimize it alternately with the rendering results of 3DGS. In this way, without changing the structure or input format of 3DGS, the denoising network can gradually adapt to three-dimensional geometric constraints and improve the final synthesis quality in noisy, sparse view scenes.

### 2.2. Image Denoising

During real-world imaging, sensors are subject to multiple factors including photon scattering, thermal noise, ISO noise from gain amplification, compression artifacts, minor jitter, motion blur, and outdoor weather conditions. These factors cause image texture to be obscured and result in brightness and color drift, leading to poor quality in 3D reconstruction tasks [10,30,31,32]. Researchers have systematically investigated noise suppression without sacrificing detail. The objectives extend beyond merely achieving higher PSNR and SSIM scores to encompass structural fidelity, cross-domain generalization, and adaptability to real-world noise. Methodologically, existing denoising approaches primarily fall into supervised and unsupervised categories. In multi-view tasks, noise and exposure variations amplify reprojection errors. Ignoring geometric consistency often leads to appearance drift and texture discontinuities in subsequent reconstructions.

Supervised image denoising methods were pioneered by BM3D [12], which established the foundation through similarity block grouping and 3D transform domain cooperative filtering. With the rise of deep learning, DnCNN [13] modeled noise as residuals, employing end-to-end convolutional networks to achieve results on synthetic Gaussian and real noise data that significantly outperformed traditional approaches. FFDNet [14] employed noise level maps and downsampled sub-maps to accelerate denoising while enhancing accuracy, advancing the practicality of a single model adapting to varying noise intensities. With the advent of the Transformer era, SwinIR [33] and Restormer [34] leveraged efficient attention mechanisms to further improve PSNR and SSIM in real-world noise scenarios. Concurrently, NAFNet [35] was ingeniously designed to deactivate nonlinear activations, reducing computational costs while maintaining or improving accuracy. Overall, supervised methods exhibit stable, convergent, and predictable performance under sufficient data and controllable noise distributions, but they often rely on annotated datasets. When camera imaging is complex and noise is non-Gaussian and time-varying, generalization and robustness remain bottlenecks for engineering implementation.

To overcome reliance on clean annotations, Noise2Noise (N2N) [15] stands as one of the pivotal early works in unsupervised methods. N2N demonstrates that networks can learn noise expectations and converge toward near-clean images when provided with paired, noise-independent, identically distributed noisy images. However, the primary limitation of N2N lies in the need to collect paired observations with distinct noise realizations. In real-world 3D reconstruction scenarios, particularly when capturing sparse viewpoints, often only one image exists per viewpoint. It is impractical to collect a second image with a different noise version for each viewpoint as a pseudo-label, making N2N difficult to apply directly. With the emergence of Noise2Void [16], which requires no paired data compared to N2N, its blindness mechanism prevents the network from simply replicating noise, making it more robust to uncertain noise patterns. More importantly, in sparse viewpoint scenarios, the single image per viewpoint fails to satisfy the dual-sample constraint of N2N. Consequently, under settings where only a single observation image exists per viewpoint and paired noisy samples cannot be obtained, N2V proves more suitable than N2N for the sparse viewpoint scenarios considered in this work.

Most of the aforementioned supervised and unsupervised denoising methods operate independently on single or small numbers of images, lacking tight coupling with the 3D geometry and multi-view rendering process. They typically assume independence between input images, failing to leverage camera poses, viewpoint distributions, or 3D scene priors to guide denoising. Furthermore, the self-supervised approach of N2V can be combined with InstantSplat to generate denoised images from noisy ones for new viewpoints after training—an advantage difficult for N2N to achieve.

### 2.3. Sparse View 3D Reconstruction

In traditional 3D reconstruction, COLMAP is typically employed to estimate camera poses and reconstruct scene point clouds [36]. It is a mature Structure from Motion (SfM) and Multi-View Stereo (MVS) system that first performs feature extraction and matching. It then obtains sparse point clouds from both inside and outside the camera through geometric validation, RANSAC [37] estimation, and incremental or global pose solving. Finally, it generates dense reconstructions using methods such as PatchMatch Stereo [38]. The strengths of COLMAP lie in its rigorous geometric inference process, high interpretability, and robust engineering ecosystem. However, under sparse viewing conditions—such as when only a few or a dozen images are available as input, or in scenarios with weak textures, repetitive patterns, low illumination, or heavy noise—sufficient usable feature matching pairs are often scarce. Incremental initialization is prone to being undermined by mismatches, leading to unstable pose maps and difficulties in correcting scale and drift. This, in turn, causes large gaps or excessive smoothing in the disparity estimation during the reconstruction phase. To address the bottleneck of matchability under sparse view and degraded conditions, the representative recent work Dense Unconstrained Stereo 3D Reconstruction (DUSt3R) [7] has made significant contributions.

DUSt3R is an end to end 3D reconstruction method for sparse multi-view data. It argues that traditional 3D reconstruction, which decomposes the task into multiple submodules such as feature extraction, feature matching, triangulation, pose optimization, and dense reconstruction, leads to error propagation across stages and makes it difficult for subsequent steps to impose constraints on earlier results. To address this, DUSt3R directly performs dense 3D prediction on uncalibrated image pairs lacking explicit pose and intrinsic parameters. It recovers 3D point positions in the camera coordinate system from an unconstrained image collection, then completes tasks such as camera calibration, depth estimation, and camera pose solution based on this foundation. The entire process relies on data-driven neural networks to directly regress 3D point clouds and correspondences from 2D image pairs. This approach bypasses intermediate steps found in traditional methods [39], such as feature point detection, feature matching, and explicit triangulation, compressing the reconstruction workflow into two core components: 3D point cloud estimation and global alignment.

Compared to classical SfM and MVS pipelines represented by COLMAP, DUSt3R demonstrates greater robustness under sparse view conditions characterized by sparse features, repetitive textures, or small baselines. It no longer relies heavily on manual features and multi-stage pipelines, thereby exhibiting superior adaptability to challenging scenarios. Building upon this foundation, InstantSplat further uses the DUSt3R network as a replacement module for COLMAP. It uses this network to estimate dense 3D points and relative camera poses from a small number of input views, serving as the geometric prior and initialization for 3DGS optimization. Consequently, even in scenarios where COLMAP frequently fails—such as those with extremely few views, weak textures, or noisy environments—InstantSplat can still obtain relatively reliable poses and initial geometry, providing a more stable foundation for subsequent Gaussian splatter training and rendering.

## 3. Methods

To address the suboptimal reconstruction performance of 3DGS in sparse view and noisy scenes, this paper proposes a method named Denoise-GS. The overall approach combines unsupervised image denoising techniques with explicitly differentiable 3DGS. The overall training process consists of two rounds: In the first round, Noise2Void (N2V) is used to train UNet for denoising. The denoised images are then processed through InstantSplat to initialize, train, and render 3DGS. The rendered results are subsequently grouped and filtered by viewpoint to ensure the representativeness and stability of the data for the second round. In the second round, the same UNet is fine-tuned using a composite loss that balances low-frequency consistency and perceptual detail consistency. Rendering is then performed again on the selected viewpoints, ultimately generating new views that achieve denoising effects. The workflow and corresponding subsections are arranged as follows: we first cover the 3DGS formulation and prerequisite knowledge, then describe the training and inference process of N2V-UNet, next we discuss pose-based viewpoint grouping and representative viewpoint selection, and finally present the formulation and optimization strategy of the second-round joint loss.

### 3.1. Preliminary

Three-dimensional Gaussian splatting represents a scene as a set of differentiable three-dimensional Gaussian primitives. Each primitive is parameterized by position, anisotropic scale with a covariance matrix, opacity, and color. In contrast to integral formulations based on volumetric rendering, 3D Gaussian splatting performs splatting and alpha compositing in screen space. Each three-dimensional Gaussian is first projected through the camera onto the image plane to produce an elliptical kernel, and the kernel strength is controlled by opacity and color. These kernels are then composited from near to far so that closer Gaussians contribute first and the contributions of farther Gaussians are attenuated by the transmittance of those in front. This process can be viewed as a finite support weighted synthesis in screen space, where correct depth ordering and visibility are essential. The projected coverage weight is obtained by projecting the *i*th three-dimensional Gaussian with center $\mu_{i}$ and covariance $\Sigma_{i}$ onto the image plane and by applying a first order approximation using the projection Jacobian *J* evaluated at $\mu_{i}$ to compute the coverage at pixel *p*:

(1) $$w_{i}\left(p\right)=\alpha_{i}\;\exp\;\left(-\frac{1}{2}{\left(p-u_{i}\right)}^{⊤}{\left(J_{\pi }\;\Sigma_{i}\;J_{\pi }^{⊤}\right)}^{-1}\left(p-u_{i}\right)\right),\;u_{i}=\pi \left(\mu_{i}\right)$$

Under this representation, the pixel color is obtained by forward compositing of several visible Gaussians in screen space. Kernels near the projection center contribute more, and the contributions of distant Gaussians are gradually attenuated. This cumulative process encodes occlusion through the recursion of transmittance, which ensures that the foreground takes effect first and that the background transitions smoothly. It maintains stable depth ordering and visibility across multiple views. It describes the correspondence from geometry to imaging. Pixels that are closer to the projection center $\mu_{i}$ receive stronger coverage from the *i*th Gaussian. The covariance transformed by the Jacobian *J* determines the shape and the scale of the kernel on the image plane. After sorting visible Gaussians from near to far by depth, the fraction not occluded by previous Gaussian esllipses up to the *i*th one is as follows:

(2) $$T_{i}\left(p\right)=\prod_{k<i}\left(1-w_{k}\left(p\right)\right)$$

The cumulative transmittance reflects the visibility along the ray from the camera to the current Gaussian. If the depth ordering or the visibility test is incorrect, the pixel residual will be misattributed to front and back layers, which causes local color shifts and blurry edges. Geometry and appearance are therefore not two independent problems. Correct ray attribution directly affects the direction of parameter updates. This expresses the physical meaning of forward compositing. Gaussians that are closer to the camera take effect first and attenuate the visibility of those behind. Hence the rendered color at pixel *p* is as follows:

(3) $$\hat{I}\left(p\right)=\sum_{i}T_{i}\left(p\right)\;w_{i}\left(p\right)\;c_{i}$$

Here $w_{i}$ is the coverage weight of the *i*th Gaussian at pixel *p*, $c_{i}$ is its color, and $T_{i}$ is the cumulative transmittance up to the *i*th Gaussian. This expression encodes the physical intuition that a closer contributor has a larger influence. During training, the primary objective is the photometric difference between the supervision image and the rendered image, and the camera extrinsics and the Gaussian parameters can be optimized jointly. It is worth noting that the projection Jacobian *J* describes the impact of camera intrinsics, camera extrinsics, and depth on the scale and the shape in screen space. The same three-dimensional structure corresponds to elliptical kernels with different shapes and orientations under different viewpoints, which is an important source of cross view consistency considerations. When the camera baseline is large or the surface normal changes rapidly, relying only on single view appearance can be ambiguous. Multi-view constraints and rendering feedback provide additional information in such cases. In the training stage, the photometric difference between the rendered image and the supervision image is the main driving force. The Gaussian center positions, shape scales, opacities, and colors are optimized together, and joint updates with the camera extrinsics can further reduce reprojection error, which clarifies depth ordering and occlusion boundaries. These preliminaries provide the necessary background and intuition for the subsequent two round closed loop.

In real-world captured images, both shot noise which varies with signal intensity and readout noise inherent to the imaging chain are present. Weak textures and low illumination further amplify these uncertainties, manifesting as high-frequency graininess and subtle color jitter. Training directly on these noisy images may cause the model to over-deblur details with larger kernels or absorb random noise into color parameters, leading to cumulative texture drift and hue shifts across viewpoints. This paper trains without clean annotations. In the first round, we introduce Noise2Void (N2V) denoising based on the blind spot concept, enabling the network to recover expected values using neighborhood information without directly observing central pixels. InstantSplat training and rendering produce multi-view images aligned with the input, providing more reliable viewpoint selection for the second training round.

### 3.2. N2V-UNet for Unsupervised Denoising

In 3D reconstruction, particularly when dealing with sparse viewpoints and lacking clean annotations, relying directly on traditional supervised learning methods for denoising and defogging often leads to severe overfitting issues. This occurs because sparse viewpoints result in incomplete texture information within the input images, and the presence of noise or fog further exacerbates this incompleteness. To address this challenge, this study adopts the Noise2Void (N2V) method as its foundation. N2V employs a self-supervised learning-based occlusion mechanism that leverages intra-image consistency for denoising. Its core principle involves randomly obscuring certain pixels within an image and reconstructing them using contextual information from surrounding pixels, without relying on any external clean image labels. Specifically, during N2V training, input images undergo occlusion processing. The network then predicts the values of occluded pixels based on the remaining pixel information, thereby achieving denoising. Through this approach, the network acquires robust image restoration capabilities, particularly suited for images with high noise levels.

**Figure 2:**
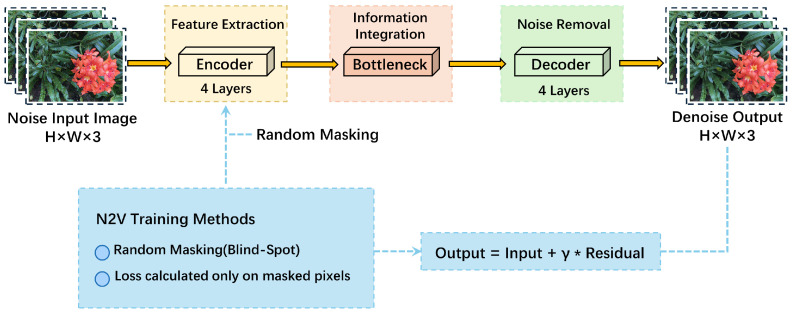
The overall network architecture diagram of N2V-UNet from input to output.

UNet is a classic encoder–decoder network architecture [17], originally developed for medical image segmentation but equally effective in tasks like image denoising. Its structural hallmark consists of symmetrical encoder and decoder modules: the encoder progressively extracts features from the input image while compressing information, while the decoder restores these feature maps to the original image dimensions through upsampling. During this process, the skip connections in UNet enable direct transmission of low-level detail information to the decoder, aiding in the recovery of high-frequency image details. This architecture proves particularly effective for tasks requiring simultaneous preservation of image detail and global structure, such as denoising and image restoration. In this study, UNet serves as the foundational architecture for the N2V network, leveraging its efficient feature propagation and detail retention capabilities to achieve noise removal while preserving image edges and textures to the greatest extent possible.The N2V-UNet framework is shown in Figure 2.

In this paper, N2V defines the unsupervised training method, while the actual pixel prediction is performed by UNet. The UNet used in this paper adopts a four-level encoder–decoder structure. The encoder consists of four convolutional blocks that progressively extract features, and at each level, the spatial resolution of the feature map is halved through downsampling to expand the receptive field and aggregate a larger context of information. The bottleneck layer is located at the lowest resolution, and it integrates global structural information. The decoder restores the resolution through four upsampling layers, and at each level, the upsampled features are concatenated with the corresponding encoder outputs through skip connections, then fused by convolutional blocks. This design allows the shallow texture and edge information to be combined with deep structural information for denoising prediction.

As a result, the N2V masking training mechanism and UNet’s multi-scale feature fusion work together, enabling the model to suppress noise while reducing the issue of excessive smoothing of details in the unsupervised setting. In our method, we combine the N2V blind spot mechanism and the encoder–decoder structure of UNet to form a complete denoising network. N2V provides an unsupervised training method by defining the training objective through pixel masking and neighborhood prediction. UNet is responsible for feature learning and pixel recovery. During training, N2V generates input images with masked pixels and calculates the loss only at the masked locations, while UNet recovers details from the neighborhood using its skip connections, learning the noise pattern and avoiding simple copying of the input. The collaboration between the two allows the model to output the complete denoised result during inference, preserving UNet’s ability to recover texture while benefiting from N2V’s advantage of learning noise statistics in an unlabeled environment, ensuring stable performance under sparse viewpoints and high noise conditions.

N2V training is performed by randomly masking pixel locations in the image and replacing the masked pixels with randomly permuted pixels from the image, creating new input images to train the network. A subset of pixels in the original image is masked during each training step, forming a mask. Then, the image with the mask applied is fed into the network for prediction. The loss function of this process is computed only at the masked pixels, and the network weights are updated by minimizing the error between the predicted and original images. In N2V training, we use a fixed-proportion random masking strategy. Specifically, for each training patch, a mask *M* is independently generated with probability *p*, and the masked pixels are replaced with randomly permuted pixel values to construct blind spot inputs. In this paper, the masking probability is set to p=0.04, meaning that 4% of the pixels are used as blind spots in each forward pass for self-supervised training. The loss is computed only at the masked positions to avoid the network simply copying the input pixels and encourage it to use the context for prediction.

The following describes the specific mathematical formula. First, construct the masking to corrupt the input image:

(4) $$\tilde{x}=x⊙\left(1-M\right)+\pi \left(x\right)⊙M$$

Here, *M* is the mask indicating which pixels are occluded, and π(x) is the random permutation of the image. Then, the output network $\tilde{x}$ is the sum of the input image and the residual predicted by the network:

(5) $$\hat{x}=\mathrm{clip}\left(x+\gamma \;r_{\theta }\left(x\right),\;0,1\right),\;\gamma \in \left(0,1\right]$$

The randomness of the masking pattern prevents the network from memorizing any single pixel. The residual fusion coefficient γ controls the trade off between the predicted residual and the original observation. A smaller γ is more conservative, and a larger γ emphasizes detail recovery. We set γ as a fixed constant of 0.5, which both controls the denoising intensity and avoids excessive smoothing. This design allows the model to remove noise while preserving the structural information of the original image, thereby helping to improve the stability of subsequent three-dimensional reconstruction. During inference, the network performs a forward pass on the input and outputs a denoised result. This result serves as the starting point for the first round rendering and the second round joint optimization, which forms a closed loop in the full pipeline. Finally, the loss is computed only at the masked pixel locations:

(6) $$L_{N2V}\left(\theta \right)=\frac{1}{|\Omega_{M}|}\sum_{\left(i,j\right)\in \Omega_{M}}{∥\;f_{\theta }{\left(\tilde{x}\right)}_{ij}-fx_{ij}\;∥}_{2}^{2}$$

This ensures that the denoising model focuses on utilizing local context to recover occluded pixels during training, rather than simply replicating known information in the image. Through this training mechanism, the N2V strategy guides the UNet to learn noise patterns by leveraging the image’s inherent spatial correlations—even without clean labels—thereby achieving effective denoising results under high-noise conditions. Throughout the N2V-UNet training process, the residual-based output encourages the model to prioritize recovering high-frequency details without compromising the original low-frequency structure and overall brightness of the image. This is crucial for maintaining cross-image consistency under sparse view conditions. During inference, N2V-UNet performs multiple random occlusions and forward passes on the same input image, averaging the predictions to further reduce variance introduced by random occlusions and mitigate potential instability in the denoising process.

### 3.3. Pose Grouping and Representative View Selection

In the second training round, to ensure the reliability of input images in terms of geometric coverage and noise characteristics, we selected a set of representative viewpoints from the first round’s rendering results as inputs for fine-grained denoising and reconstruction. Given significant variations in baseline, orientation, and noise levels across different viewpoints, directly using all rendered frames risks introducing visual inconsistencies, amplified exposure differences, and heightened noise propagation. Therefore, it is essential to first perform a structured selection of viewpoints at the pose level.

In the implementation, we first obtain the geometric information for each frame based on the camera extrinsic parameters output by InstantSplat. Given the rotation matrix $R_{n}$ and translation vector $t_{n}$, the camera center can be expressed as $c_{n}=-R_{n}^{T}t_{n}$, while the forward direction is derived from the third column of the rotation matrix, denoted as $z_{n}=R^{T}{\left[0,0,1\right]}^{T}$. Combining these two components into a six-dimensional vector $\left[c_{n},z_{n}\right]$ simultaneously describes the spatial position and imaging direction for that viewpoint. We perform k-means [40] clustering in the 6-dimensional feature space to group the viewpoints, with the number of clusters fixed at K=12, which matches the number of input viewpoints required for the second round of training. k-means uses $\left[c_{n},z_{n}\right]$ as the clustering input and iteratively minimizes the Euclidean distance within each group, ensuring that viewpoints with similar camera center positions and forward directions are grouped together. This clustering method does not require manually setting distance thresholds or relying on scene-specific parameters. It is stable and computationally efficient, while also ensuring that viewpoints within the same group remain relatively consistent in terms of line of sight direction and baseline, making the images within the group more comparable geometrically. After the grouping, we calculate the Laplacian variance within each cluster to measure sharpness and select the frame with the highest score from each cluster as the representative viewpoint. This way, we obtain 12 representative frames from the candidate set for the second round of denoising and reconstruction.

(7) $${Var}_{Lap}\left(I\right)=Var\left(\nabla^{2}I\right)$$

Here, $\nabla^{2}I$ denotes the Laplacian gradient of an image, whose variance reflects the richness of high-frequency details within the image. Higher values indicate more pronounced texture and edge information. Within the same pose neighborhood, we select the frame with the highest Laplacian variance as input for the second round of UNet training. This prevents blurry or heavily noisy images from entering subsequent training stages. This strategy ensures the network receives more stable images that align with geometric relationships, enhancing consistency during optimization. Since images with higher Laplacian variance typically exhibit more pronounced detail representation, they provide more reliable reference quality for the second round. Finally, we select the sharpest image from each group as the representative viewpoint. These viewpoints form the input images, ensuring comprehensive perspective coverage while eliminating frames with significant noise or blur. Input scale is controlled to reduce storage and computational overhead. Through this grouping and representative viewpoint selection process, our method preserves essential geometric variations while reducing noise interference, providing a more robust input source for subsequent denoising and reconstruction.

### 3.4. Joint Loss

The objective of the second round is to refine the first round denoised images, enhancing detail sharpness and texture fidelity while preserving structural consistency. We adopt a composite objective that balances low frequency agreement with high frequency fidelity.

(8) $$L\left(\theta \right)=\alpha \;L_{coarse}\left(\theta \right)+\left(1-\alpha \right)\;L_{fine}\left(\theta \right),\;\alpha \in \left[0,1\right]$$

The low frequency consistency term compares the input and the output after smoothing. It focuses on preserving tone and large scale structure, and it prevents damage to the global appearance during refinement. The detail consistency term consists of a pixel level robust distance, a structural similarity term, a gradient consistency term, and a chroma consistency term. The robust distance suppresses the influence of outlier noise. The structural term encourages alignment of textures and contours. The gradient term emphasizes the match of edge direction and edge strength. The chroma term reduces color shifts caused by viewpoint changes. The composite perceptual loss considers both low frequency consistency and detail consistency in order to achieve a balanced result. The low frequency consistency loss aims to preserve the overall structure and the overall tone of the image, and it avoids excessive correction of global characteristics during training. To this end, we apply Gaussian smoothing to the input image and to the output image, and then compute their difference. Here ρ denotes the Charbonnier loss [41], which is more robust to outliers and prevents excessive smoothing of the low frequency part.

(9) $$L_{coarse}=\frac{1}{\left|\Omega \right|}\sum_{p\in \Omega }\rho \;\left(G_{\sigma }\left(\hat{x}\right)\left(p\right)-G_{\sigma }\left(x\right)\left(p\right)\right),\;\rho \left(\delta \right)=\sqrt{\delta^{2}+\varepsilon^{2}}$$

The detail consistency loss focuses on preserving image details, textures, and edges. To avoid over sharpening during detail restoration, the loss consists of several sub-terms. The first is a pixel level robust distance. The second is structural similarity (SSIM) [42] that measures structural agreement. The third is gradient consistency that preserves edges. The last is chroma consistency that corrects color bias. The expression is given below.

(10) $$\begin{array}{cc} L_{fine}= & \lambda_{1}\;\rho \left(\hat{x}-x\right)+\lambda_{2}\;\left(1-\mathrm{SSIM}(\hat{x},fx)\right)+\lambda_{3}\;∥\nabla \hat{x}-\nabla x∥\\ \; & \;+\lambda_{4}\;∥\mathrm{Chr}\left(\hat{x}\right)-\mathrm{Chr}\left(x\right)∥+\lambda_{5}\;\mathrm{TV}\left(\hat{x}\right) \end{array}$$

These sub-terms preserve edge structure, reduce color discrepancy, and avoid over sharpening. The coefficient $\lambda_{1}$ controls overall pixel fidelity, $\lambda_{2}$ enforces structural similarity, $\lambda_{3}$ strengthens detail through the gradient loss, $\lambda_{4}$ corrects color consistency, and $\lambda_{5}$ suppresses unnecessary high frequency noise through total variation regularization. With this training strategy, the second round performs fine deblurring while maintaining global structural consistency, and it significantly improves image detail and clarity, which leads to high quality three-dimensional reconstruction. Within this framework, the total variation regularizer acts as a balancer. It limits high frequency oscillation without over weakening true edges, and together with the pixel level robust distance, it suppresses the reappearance of noise. In the second round of training, these sub-terms jointly constrain the relation between the denoised output and the rendered result, so that appearance details gradually become consistent under geometric reference. Over reliance on a single term may cause loss of detail or color shift, therefore the coefficients should be numerically balanced to trade off sharpness and stability. The final outputs show less flicker and more coherent textures in novel view synthesis while remaining consistent with the supervision images. Building on the appearance baseline from the first round, the joint perceptual loss in the second round helps the network improve steadily in detailed regions and transfers the gains to the three-dimensional rendering.

The proposed method Denoise-GS includes unsupervised denoising with an N2V-UNet, pose-based grouping and selection of representative viewpoints, and the use of a joint perceptual loss in the second round of training. With this layered and progressive optimization strategy, the method improves three-dimensional reconstruction quality under very sparse viewpoints and without clean labels. The approach is theoretically sound and also scalable and stable in practice.

## 4. Experiments

To evaluate the performance of the proposed method for reconstruction in sparse view noisy scenes, we conduct experiments on the public LLFF [43] dataset. To simulate noisy conditions, Gaussian noise is added to the input images. We compare our approach with representative reconstruction methods, including 3DGS [1], Mip-Splatting [23], Scaffold-GS [44], and InstantSplat [6], and perform a series of ablation studies to analyze the contribution of each key component. Finally, we provide a discussion of the method and identify its limitations.

### 4.1. Experimental Setup

All experiments are conducted on a workstation equipped with an i9 13900K CPU, an NVIDIA GeForce RTX 4080 GPU, and 128 GB of RAM. The CPU has a maximum clock frequency of 5.8 GHz, and the GPU provides 16 GB of GDDR6X memory and 10,240 CUDA cores.

To accurately evaluate the quality of reconstruction and rendering, we compare the differences between the images rendered by the algorithm and the ground truth images under different camera poses. The evaluation metrics include peak signal to noise ratio (PSNR), the Structural Similarity Index Measure (SSIM) [42], and the Learned Perceptual Image Patch Similarity (LPIPS) [45]. In the joint loss of the second stage, we set α to 0.35, which yields the best performance. In $L_{\mathrm{fine}}$, we set $\lambda_{1}=0.25$, $\lambda_{2}=0.5$, $\lambda_{3}=0.15$, $\lambda_{4}=0.05$, and $\lambda_{5}=0.05$ in the experiments. Before training, the number of input images for our method was set to 12. The inputs for other baseline methods are also 12 images of different poses.

### 4.2. Basic Comparison

**Figure 3:**
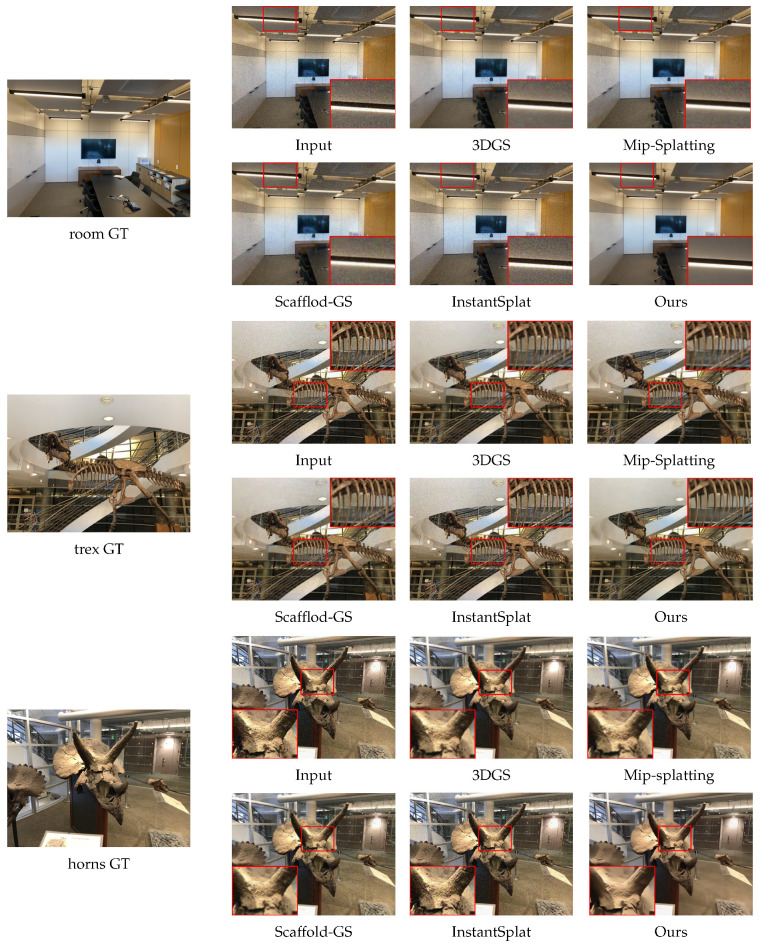
Overview of denoising results. This figure compares our method with other advanced reconstruction approaches on the LLFF dataset with added noise. The image shows a detailed comparison of magnified sections. It is evident that our method significantly outperforms others in terms of clarity and denoising quality.

We conduct comparisons on the LLFF dataset with 3DGS, Mip-Splatting, Scaffold-GS, and InstantSplat. To simulate real imaging degradation, we add Gaussian noise to the input images, with the noise level set to σ=0.25. The main evaluation metrics are peak signal to noise ratio (PSNR), structural similarity index (SSIM), and learned perceptual image patch similarity (LPIPS). For 3DGS, Mip-Splatting, Scaffold-GS, and InstantSplat, we use the same image resolution, the same number of training views, and the same number of iterations. For our method, we use sparse viewpoints and fewer training iterations. We perform experiments on three scenes, each scene was tested three times, and the metrics are reported as mean ± standard deviation. The results are shown in Figure 3.

From the comparison of the test results across different scenes, it can be observed that when input images with significant noise are provided, existing state-of-the-art reconstruction methods still leave granular noise in the reconstruction and show blurring at the edges. In contrast, our method performs better in noise suppression and clarity. The evaluation metrics are shown in Table 1. Our method shows improvements in both PSNR and SSIM, while the LPIPS metric is significantly reduced.

Overall, our method shows significant improvements in reconstruction when handling noisy images. This is primarily due to our comprehensive strategy in data selection and objective design. We reduce ineffective inputs through pose-based photo selection and adopt a coarse to fine joint loss during the training phase to coordinate the denoising and reconstruction processes, ensuring stable performance under varying noise intensities and viewpoint configurations. In general, the method achieves a more balanced handling of details and noise, with more consistent results across different scenes and a more natural appearance. In multiple experiments, the method reduces texture fragmentation, avoids over smoothing in flat regions, and ensures more stable boundary transitions. Even with limited input data or strong noise, the method still maintains its advantages.

### 4.3. Ablation Study

**Figure 4:**
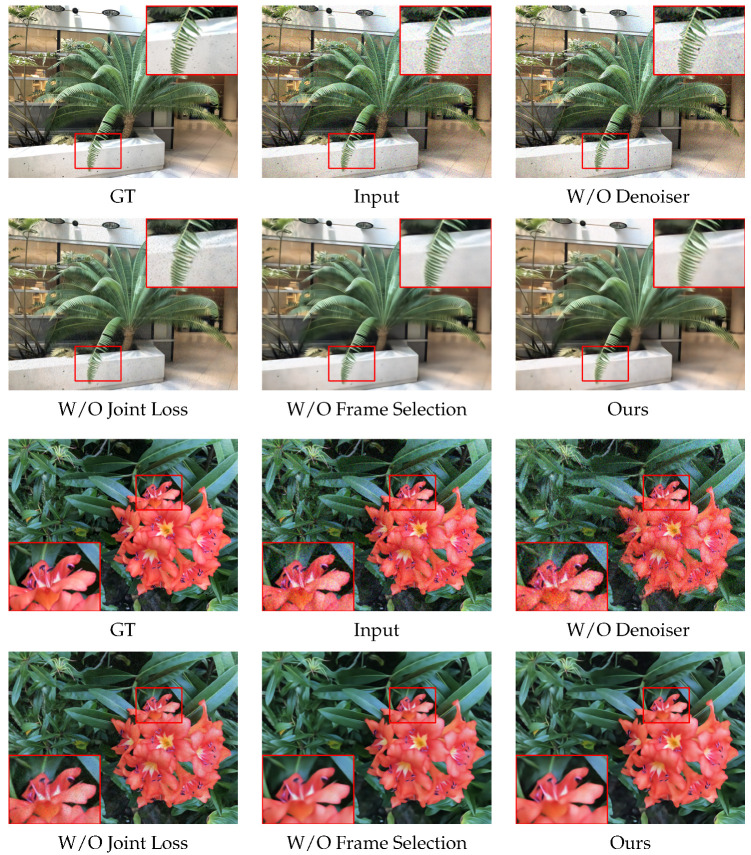
Overview of the ablation study. This section examines the impact of each component of the proposed method on the experiment in two representative scenes.

To systematically evaluate the contribution of each key design to the overall performance, we conduct an ablation study. Specifically, the study focuses on three core components: the impact of joint denoising using UNet in the first and second rounds on reconstruction quality, the effect of the pose-based image selection mechanism, and the influence of the second stage joint loss on reconstruction quality. After performing the ablation study on two scenes of the LLFF dataset, the reconstruction comparisons are shown in Figure 4. From the figure, it can be observed that without the two-round UNet denoising, the reconstructed images still contain noise, especially in regions with sparse texture, where noise is more apparent. In contrast, our complete approach effectively removes the noise and improves the reconstruction quality.

In the second stage, we conducted experiments where the joint weighted loss was removed and replaced with the Charbonnier loss. The results show that, after removing the joint weighted loss, the reconstructed images still contain some residual noise, which was not completely eliminated. In comparison, our complete approach better removes the noise present in the input images. The results of the experiment where the frame selection strategy was removed are similar. The reconstructed images have lower quality compared to the full version of the method, and some noise is still present.

We conducted experiments on the fern and flower scenes from the LLFF dataset. Each scene was tested three times, and the metrics are reported as mean ± standard deviation. The performance comparison metrics are shown in Table 2. In the flower scene, both PSNR and SSIM metrics show a significant increase, while the LPIPS metric decreases. In the fern scene, PSNR and SSIM also show an increase, while the LPIPS metric shows a slight increase. In the frame selection module, we first group the images based on their poses, and then evaluate the image clarity using Laplacian variance within each group. The image with the highest score is selected as the input image. The image with the largest Laplacian variance is typically the sharpest, but sharpness does not necessarily equate to structural consistency. For scenes like the fern, where high-frequency textures are rich, especially with the sharp details in the fern leaves, the viewpoint with the largest Laplacian variance may lead to some local textures or details becoming too abrupt, which affects the SSIM and LPIPS scores. This is a limitation of our design, as we aim to achieve a good balance between denoising and retaining detailed textures. However, this does not affect the robustness of our method when handling noisy three-dimensional reconstruction scenes.

### 4.4. Training Rounds

To evaluate the impact of training rounds on reconstructed image quality, we increased the number of training rounds from two to four. Each experiment was conducted three times, and the PSNR are reported as mean ± standard deviation. The training results are shown in Table 3, which also records the PSNR metrics and computational time after completing 30,000 iterations per round. The experimental results demonstrate that two rounds of training already yield significant improvements. The PSNR for the first round was 29.39 dB. After adding a second round of refinement, the PSNR improved to 30.60 dB, demonstrating a noticeable overall quality increase while keeping the training time within a reasonable range. However, adding a third and a fourth training round does not bring further benefits. The PSNR in the third round decreases to 28.65 dB, which is even lower than the first round. This occurs because the model attempts stronger noise suppression in this round, removing some high frequency details and temporarily reducing image quality. In the fourth round, PSNR increases slightly to 30.94 dB, but the improvement is minimal. The model adjusts the balance between noise suppression and detail preservation, which leads to a small rise in PSNR. This behavior reflects the trade off between denoising and detail recovery during training. However, it also introduces more than 30 min of additional training time.

In summary, a two-round denoising and reconstruction loop is sufficient to stabilize performance. Increasing the number of stages does not significantly improve PSNR, adds unnecessary time cost, and may even degrade image quality. Therefore, we adopt two stages as the default setting to balance performance and efficiency.

### 4.5. Discussions and Limitations

Based on the combined experimental results, our method achieves superior reconstruction performance in noisy scenes compared to current mainstream approaches such as 3DGS, Mip-Splatting, Scaffold-GS, and InstantSplat. This advantage stems primarily from our joint weight loss function and image filtering mechanism. The former constrains low-frequency consistency and high-frequency details, enabling the model to achieve higher reconstruction quality with fewer iterations. The latter enhances the coverage of effective parallax and filters out detrimental input data. In ablation studies, the LPIPS metric performed less favorably in the fern scene, which we attribute to its fragmented random textures; however, other metrics remained relatively stable. Secondly, increasing the overall number of training epochs does not effectively improve PSNR in reconstructed images while significantly increasing computational time. From the above experimental results, we conclude that our method has some limitations: in areas with dense textures, reconstruction may cause slight smoothing artifacts in the images. During the second optimization round, ultra-high-resolution large scenes may incur substantial GPU memory and computational overhead. Nevertheless, our method demonstrates superior robustness across diverse scenarios, achieving higher reconstruction quality and more consistent subjective perception with fewer iterations.

## 5. Conclusions

This paper addresses the challenge of 3D reconstruction under noisy and low viewpoint conditions by proposing a denoising 3DGS framework. It employs pose-guided photo selection to enhance effective disparity coverage while suppressing detrimental inputs. During two rounds of denoising, a coarse-to-fine joint loss simultaneously constrains low-frequency consistency and high-frequency detail. Comparative experiments across diverse LLFF datasets demonstrate superior performance compared to mainstream methods such as 3DGS, Mip-Splatting, Scaffold-GS, and InstantSplat. Our approach achieves higher PSNR and SSIM while maintaining lower LPIPS in most scenarios, converging stably with fewer iterations. Ablation studies further validate that the proposed camera-pose-based image selection mechanism and joint loss are pivotal for reconstruction performance and stability. Although our method exhibits slight oversmoothing in highly textured scenes, it effectively removes noise from input images, significantly reduces graininess in flat areas, and enhances texture–edge coherence. Overall, this work achieves a favorable balance among robustness, detail fidelity, and computational overhead. To address the aforementioned limitations, future work will advance in several directions. First, to mitigate the slight oversmoothing in densely textured regions, we will further investigate detail preservation and cross-view consistency constraints related to texture intensity, adapting the balance between denoising and detail retention in the joint weights. Second, to reduce memory and computational demands for ultra-high-resolution large scenes, we will evaluate more resource-efficient strategies such as batch training and hierarchical resolution scheduling, aiming to lower costs without compromising convergence stability. In addition, we will conduct systematic evaluations of noise distributions across different acquisition devices and shooting conditions, and model real sensor noise to explore reconstruction tasks under more complex and realistic noise scenarios.

## Figures and Tables

**Table 1:** Quantitative comparison of our method with state-of-the-art methods using three metrics across three scenes. Our method achieves higher PSNR and SSIM compared to other baseline methods, while also achieving the lowest LPIPS values.

**Method**	**Room**		
	**PSNR ↑**	**SSIM ↑**	**LPIPS ↓**
3DGS	22.46 ± 0.28	0.574 ± 0.011	0.286 ± 0.014
Mip-Splatting	23.54 ± 0.43	0.681 ± 0.012	0.296 ± 0.018
Scaffold-GS	23.25 ± 0.36	0.610 ± 0.006	0.329 ± 0.008
InstantSplat	23.28 ± 0.36	0.617 ± 0.011	0.309 ± 0.014
Ours	**33.85 ± 0.17**	**0.934 ± 0.002**	**0.060 ± 0.003**
**Method**	**Trex**		
	**PSNR ↑**	**SSIM ↑**	**LPIPS ↓**
3DGS	22.88 ± 0.20	0.645 ± 0.013	0.171 ± 0.014
Mip-Splatting	22.96 ± 0.22	0.647 ± 0.013	0.203 ± 0.016
Scaffold-GS	22.70 ± 0.19	0.608 ± 0.007	0.213 ± 0.009
InstantSplat	21.65 ± 0.21	0.527 ± 0.011	0.211 ± 0.012
Ours	**29.85 ± 0.14**	**0.912 ± 0.002**	**0.093 ± 0.004**
**Method**	**Horns**		
	**PSNR ↑**	**SSIM ↑**	**LPIPS ↓**
3DGS	18.42 ± 0.75	0.478 ± 0.048	0.274 ± 0.024
Mip-Splatting	18.49 ± 0.75	0.489 ± 0.050	0.358 ± 0.022
Scaffold-GS	18.35 ± 0.73	0.457 ± 0.046	0.314 ± 0.023
InstantSplat	18.35 ± 0.74	0.453 ± 0.047	0.299 ± 0.025
Ours	**28.09 ± 0.51**	**0.799 ± 0.037**	**0.200 ± 0.017**

The values in bold indicate the best results in each column.

**Table 2:** Quantitatively comparing the impact of each component of our method using three evaluation metrics on two scenes. After training with the complete model, the obtained PSNR and SSIM values are generally higher than those achieved by training with any single module, while the LPIPS values are lower.

	**Fern**		
	**PSNR ↑**	**SSIM ↑**	**LPIPS ↓**
w/o Denoiser	24.87 ± 0.31	0.622 ± 0.015	0.236 ± 0.024
w/o Joint Loss	26.48 ± 0.36	0.745 ± 0.005	0.195 ± 0.009
w/o Frame Select	27.49 ± 0.41	**0.849 ± 0.007**	**0.186 ± 0.005**
Ours	**27.52 ± 0.42**	0.847 ± 0.007	0.193 ± 0.006
	**Flower**		
	**PSNR ↑**	**SSIM ↑**	**LPIPS ↓**
w/o Denoiser	23.18 ± 0.76	0.597 ± 0.032	0.289 ± 0.038
w/o Joint Loss	27.39 ± 0.17	0.784 ± 0.006	0.198 ± 0.007
w/o Frame Select	27.93 ± 0.17	0.834 ± 0.004	0.161 ± 0.008
Ours	**28.12 ± 0.17**	**0.836 ± 0.004**	**0.160 ± 0.008**

The values in bold indicate the best results in each column.

**Table 3:** This table presents the results for PSNR and training time across different numbers of training rounds.

	PSNR	Training Time
One Round	29.40 ± 0.20	33 min
Two Rounds	30.57 ± 0.17	64 min
Three Rounds	28.63 ± 0.22	97 min
Four Rounds	30.92 ± 0.18	127 min

## Data Availability

The datasets generated and/or analyzed during the current study are available from the corresponding author on reasonable request.
